# Digestive Responses to Fortified Cow or Goat Dairy Drinks: A Randomised Controlled Trial

**DOI:** 10.3390/nu10101492

**Published:** 2018-10-12

**Authors:** Amber M. Milan, Alison J. Hodgkinson, Sarah M. Mitchell, Utpal K. Prodhan, Colin G. Prosser, Elizabeth A. Carpenter, Karl Fraser, David Cameron-Smith

**Affiliations:** 1 Liggins Institute, University of Auckland, 85 Park Road, Grafton, Private Bag 92019, Auckland 1023, New Zealand; a.milan@auckland.ac.nz (A.M.M.); sarah.mitchell@auckland.ac.nz (S.M.M.); u.prodhan@auckland.ac.nz (U.K.P.); 2 Food and Bio-based Products, AgResearch, Private Bag 3123, Hamilton 3240, New Zealand; alison.hodgkinson@agresearch.co.nz; 3 Department of Food Technology and Nutritional Science, Mawlana Bhashani Science and Technology University, Tangail 1902, Bangladesh; 4 Dairy Goat Co-operative (NZ) Ltd., 18 Gallagher Dr, Melville, Hamilton 3206, New Zealand; colin.prosser@dgc.co.nz (C.G.P.); liz.carpenter@dgc.co.nz (E.A.C.); 5 AgResearch Grasslands, Private Bag 11008, Palmerston North 4442, New Zealand; karl.fraser@agresearch.co.nz; 6 Riddet Institute, Massey University, Private Bag 11222, Palmerston North 4442, New Zealand

**Keywords:** fortified milk, goat milk, nutrition, adult, protein hydrolysis, digestion, gastrointestinal

## Abstract

Fortified milk drinks are predominantly manufactured from bovine (cow) sources. Alternative formulations include those prepared with hydrolysed bovine milk proteins or from alternate *bovidae* species, such as caprine (goat) milk. Currently, there is little data on protein digestive and metabolic responses following ingestion of fortified milk drinks. To examine the digestive and metabolic responses to commercially-available fortified milks, young adults (*n* = 15 males: 15 females), in a randomised sequence, ingested isonitrogenous quantities of whole cow-protein (WC), whole goat-protein (WG), or partially-hydrolysed whey cow-protein (HC), commercial fortified milks. Plasma amino acid (AA) and hormonal responses were measured at baseline and again at 5 h after ingestion. Paracetamol recovery, breath hydrogen, and subjective digestive responses were also measured. Postprandial plasma AA was similar between WC and WG, while AA appearance was suppressed with HC. Following HC, there was a negative incremental AUC in plasma branched-chain AAs. Further, HC had delayed gastric emptying, increased transit time, and led to exaggerated insulin and GLP-1 responses, in comparison to whole protein formulas. Overall, WC and WG had similar protein and digestive responses with no differences in digestive comfort. Contrastingly, HC led to delayed gastric emptying, attenuated AA appearance, and a heightened circulating insulin response.

## 1. Introduction

Dairy is an important source of protein throughout all stages of life [1,2]. While cow (bovine) milk is the predominant source of dairy consumed globally, alternative milk sources, including goat (caprine) milk, are also available [3]. For young children, the composition of whole cow milk may not be optimal, with more protein and fewer micronutrients (e.g., iron, vitamin D) than may be required during rapid growth and development [4,5]. This has led to the formulation of fortified milk drinks intended to supplement the diets of children above 12 months. Fortified milk drinks have been demonstrated to reduce the incidence of inadequate micronutrient intake in circumstances of unbalanced dietary intake [6,7,8], while potentially limiting the risks of excessive protein intake [4]. Fortified milk products have also emerged for adolescent or adult populations such as the elderly, offering a means of caloric regulation [9] or micronutrient and protein enrichment [10,11] of the diet.

The total protein concentration of goat milk does not differ markedly from cow milk [12], but the proportion of individual casein proteins in goat milk are more variable due to polymorphisms within the α_s1_-casein gene [13,14,15]. Low concentrations of α_s1_-casein in goat milk are associated with larger casein micelles [13,16] and lower coagulation properties of rennet curd [17,18,19]. Since different microstructures of casein gels influence pepsin diffusion and digestion [20], it might be expected that digestion rates of fortified milk drinks made from goat milk would differ from those made with cow milk. Additionally, goat milk contains greater concentrations of key essential amino acids (AA), including cysteine, tyrosine and lysine, and the branched-chain AAs (BCAAs), isoleucine and valine [3], although values reported in the literature vary [21].

Based on the available evidence, it can be hypothesised that the protein digestion of fortified milk drinks derived from either cow or goat milk will differ. It is hypothesised that due to the differing casein and coagulation properties, goat milk would be digested more rapidly [22,23] as a consequence of increased gastric transit [24]. However, this has not previously been analysed clinically. Partially-hydrolysed bovine whey fortified milks have also been proposed as alternatives to intact milk proteins in situations of heightened paediatric digestive discomfort [25]. For adults, hydrolysates are a common ingredient in supplemented protein formulations, with potential advantages for protein metabolism [26]. It can be further hypothesised that partially-hydrolysed bovine whey-fortified milk would elicit faster amino acid appearance [26] and different digestive responses on the basis of different gastric emptying, as previously reported [27]. To investigate this, the digestive responses, subsequent to the ingestion of either whole cow-protein (WC) fortified milk, whole goat-protein (WG) fortified milk, or a partially-hydrolysed whey cow-protein (HC) fortified milk product, were analysed in young adults. This included measurement of the rate of gastric emptying, rate of gastrointestinal transit, and subjective digestive comfort. Further, plasma hormonal (insulin and glucagon-like protein-1 (GLP-1)) and AA responses were measured. This study was conducted in young healthy adults, enabling the collection of both subjective reporting of digestive comfort and multiple biological samples. 

## 2. Materials and Methods

### 2.1. Subjects

**Figure 1:**
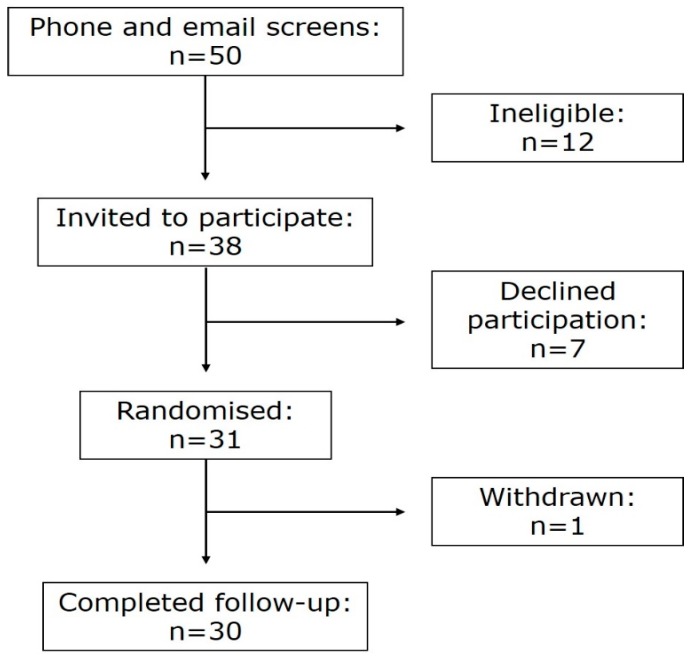
Participant eligibility, enrolment and randomisation.

A total of 31 healthy men (*n* = 15) and women (*n* = 16) aged 18–28 years were recruited to participate using digital and print advertisements. One subject withdrew prior to completion of the protocol (Figure 1). The study was conducted according to the guidelines laid down in the Declaration of Helsinki, and all procedures involving human subjects were approved by the Southern Health and Disability Ethics Committee (New Zealand, 15/STH/167). Written informed consent was obtained from all subjects. Prospective clinical trial registration was registered at www.anzctr.org.au (ACTRN12615001359527).

All participants self-reported lactose tolerance, without current or past history of gastrointestinal diseases including gastric reflux, irritable bowel syndrome, inflammatory bowel disease and Crohn’s disease, anosmia, or any regular medication use impacting digestive function (i.e., stomach acid regulators). Further, all subjects reported no metabolic or cardiovascular disease. Participants were ineligible if they had a self-reported allergy to dairy or paracetamol, or had an alcohol intake exceeding 28 units/week. No participant was engaged in athletic or extreme physical activities. All participants reported a regular consumption of dairy products. 

### 2.2. Experimental Design

A cross-over design was used with a treatment arm sequence randomly generated by www.randomizer.org [28]. The sequence allocation was concealed in sealed envelopes assigned to participants before the intervention. Investigators and participants were blinded to treatment identity for the duration of data analysis. However, no sensory masking of products was employed.

### 2.3. Study Procedures

Subjects attended the Maurice Paykel Clinical Research Unit at the University of Auckland between January and April 2016 on three occasions, separated by at least one week but no more than two weeks. The day before each visit, subjects were asked to abstain from vigorous physical exercise, to avoid foods high in fat, and to avoid fibre, lactose, fructose, and artificial sweeteners; subjects were provided guidelines for food choices based on the standard preparation for hydrogen breath testing. Subjects were asked to avoid the use of laxatives and antacids. Subjects were provided with a standardised low fat, low fibre evening meal and remained fasted from 10 pm the night prior to the intervention. 

Upon arrival, subjects were weighed (Tanita^®^ 1582 Medical Scale, Wedderburn, Auckland, New Zealand) and a venous cannula inserted for fasting blood sample collection. Further, fasting breath samples were collected and visual analogue scale (VAS) questionnaires for gastrointestinal symptomology completed. Subjects then consumed 500–900 mL of the randomised fortified milk drink including 1.5 g of paracetamol dissolved in a 300 mL portion of the milk. Fortified milk doses were calculated to provide 0.23 g/kg body weight of protein in a single serve [29], and were individualised for each subject. Recovery of plasma paracetamol was used as a proxy for gastric emptying [30].

Thereafter, blood samples were collected every 15 min until 90 min, and then hourly between 2 and 5 h. Breath samples were provided every 30 min until 5 h. 

### 2.4. Study Treatments

Whole goat-protein (WG) fortified milk and whole cow-protein (WC) fortified milk were commercially available products manufactured by Dairy Goat Co-operative (N.Z.) Limited. The partially-hydrolysed cow whey protein (HC) fortified milk was a commercial formula manufactured by Nestlé New Zealand Limited (NAN OPTIPRO^®^ HA, Germany) and purchased in New Zealand. The formulations were provided to subjects over the course of the three visits. The nutrient and total amino acid composition of each fortified milk drink are listed in Table 1 and Table 2 respectively. The powdered formulas had different nutrient contents, but were made up to provide a similar protein content in the drink. The milk solid composition of the whole protein fortified milks (WG and WC) were whole and skim milk powders, while the HC fortified milk contained only whey, with no milk fat content. The vegetable oil content of the HC fortified milk was sourced from palm, canola, and sunflower oils, while the WG fortified milk contained soyabean and sunflower oils (20% wt:wt of total fat). The WC fortified milk did not contain vegetable oils. Fructose, inulin, and oligofructose were included in the WG and WC fortified milks. Additionally, WC had added inositol.

### 2.5. Biochemical Analysis

Venous bloods were collected in ethylenediaminetetraacetic acid (EDTA) containing vacutainers (Becton Dickinson & Company, Mount Wellington, New Zealand). Plasma was prepared by centrifugation at 2000× *g* for 15 min at 4 °C and frozen at −80 °C, prior to analyses.

Plasma free amino acids were measured using ultra performance liquid chromatography (UPLC), as previously described [31]. In brief, 20 μL of plasma with internal standard L-Nor-Valine were acid extracted, then AccQ-tag reagent was added to the supernatant. UPLC was performed in singlicate using a Thermo Scientific Dionex Ultimate 3000 system (ThermoFisher Scientific, Dornierstrasse, Germany) with a Kinetex column preceded by a Krudkatcher inline filter (Phenomenex, Auckland, New Zealand). Three quality control plasma samples were included in each batch. The mean (range) overall coefficient of variation for the amino acids was 6.7% (2.7–13.9%). Data were captured by computer with Chromeleon 7.1 software (ThermoFisher Scientific, Dornierstrasse, Germany) and used to calculate amino acid concentrations from standard curves generated for each amino acid. Fortified milk total amino acids were measured by HPLC (Waters Corp., Milford, MA, USA) following 24 h hydrolysis in 6 M HCl containing 0.1% phenol, as previously described [32]. Cysteine and methionine were measured after prior preoxidation step with performic acid to form cysteic acid and methionine sulfone, while tryptophan underwent base hydrolysis prior to quantification. No correction was made for loss of AA during this hydrolysis.

Plasma glucose, and paracetamol were measured using a Cobas c311 clinical chemistry analyser (Roche Diagnostics, Basal, Switzerland) by enzymatic colorimetric assays (Roche, Mannheim, Germany). Plasma insulin was measured using the Cobas e411 immunoassay analyser (Roche Diagnostics, Basal, Switzerland) by electrochemiluminescence (Roche).

Appetite hormones (ghrelin, leptin, GLP-1) were measured in plasma using a flow cytometric multiplex array (Milliplex MAP Kit Human Metabolic Hormone Magnetic Bead Panel Assay, Millipore, MO, USA). Cholecystokinin (CCK) was measured by enzyme immunoassay (CCK (26–33) EIA, Phoenix Pharmaceuticals Inc., Belmont, CA, USA).

### 2.6. Breath Hydrogen Analysis

Breath samples were collected using the AlveoSampler™ Breath Test Kit and analysed with the BreathTracker H2+ (Quintron, Milwaukee, WI, USA). Data were recorded as CO_2_ corrected H_2_ concentrations (ppm) as a measurement of carbohydrate (lactose and inulin) malabsorption.

### 2.7. Digestive Symptoms and Appetite Visual Analogue Scales

Fasting and postprandial scores of digestive symptoms and subjective appetite were assessed using a 100 mm VAS. Severity of digestive symptoms were recorded with 0 mm corresponding to ‘no symptom’ and 100 mm to ‘the most severe symptom imaginable’. Symptoms assessed included abdominal pain/discomfort, abdominal cramps, bloating, abdominal rumbling or gurgling, flatulence, and faecal urgency. Appetite scores assessed included hunger, satisfaction, fullness, appetite, and desire to consume sweet, salty, savoury, or fatty foods, as described previously [33]. 

### 2.8. Statistical Analysis

Sample size calculations of 30 subjects were based on previously-reported peak BCAA concentration differences at 60 min of 608 μmol/L [31], with an estimated relevant difference of 20%, a standard deviation of 198 μmol/L, power of 0.9, an α of 0.05. Plasma AAs were pooled for analysis into total AAs (TAAs), essential AAs (EAA), BCAAs, and non-essential AAs (NEAA). 

Statistical analyses were undertaken using SPSS version 23 (SPSS, IBM Corporation, Armonk, NY, USA). Data are presented as means ± SEMs. Homeostatic model assessment of insulin resistance (HOMA-IR) was calculated as described previously [34]. Incremental area under the curve (AUC) was calculated after subtraction of fasting values, and was used to inform the analyte availability in circulation over the course of the postprandial period. Baseline characteristics were compared using one-factor analysis of variance (ANOVA; sex compared between subject), AUC with one-factor repeated measures ANOVA (treatment compared within subject), and three-factor repeated measures ANOVA (treatment and time compared within subject, sex compared between subject) was used for all other measures. Sidak adjusted post hoc tests were used for all multiple comparisons. The Huynh-Feldt correction was used where Mauchly’s sphericity test failed. Alpha was set at *p* < 0.05. Heat maps were created using R software version 2.15.2 with gplots (heatmap.2), RColorBrewer and colorRamps packages (R Development Core Team, Vienna, Austria).

## 3. Results

### 3.1. Demographics

Male and female subjects were equally represented in the study (*n* = 30), with no difference in mean age between sexes (*p* = 0.594). Males were taller with greater body mass index (BMI), than females (*p* < 0.001, and *p* = 0.017 respectively; Table 3). Males subjects had higher fasting glucose than female subjects (*p* = 0.001). HOMA-IR and fasting insulin were not different between sexes (*p* = 0.955 and *p* = 0.528, respectively).

### 3.2. Postprandial Amino Acid Response

**Figure 2:**
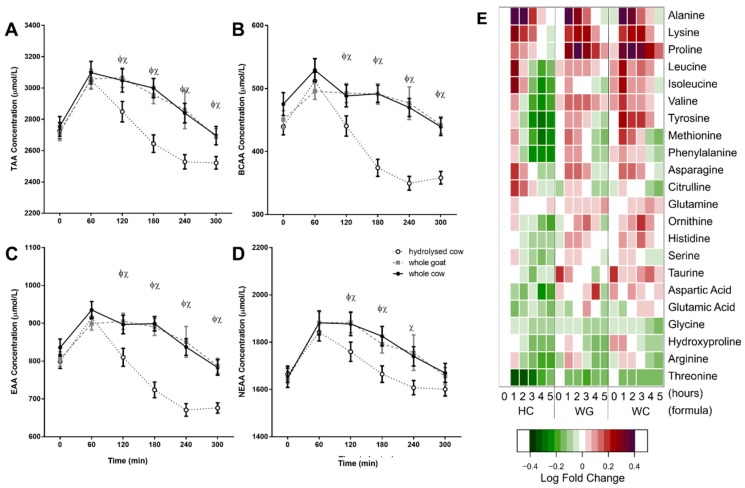
Plasma amino acid responses following formula ingestion. (**A**) Total amino acids (TAA). (**B**) BCAA. (**C**) Essential amino acids (EAA). (**D**) Non-essential amino acids (NEAA), (**E**) Individual amino acid concentrations as fold changes. Values represent means ± SEM in μmol/L (**A**–**D**) for whole cow (WC; ●), hydrolysed cow (HC; ○), and whole goat (WG; ■) fortified milks and mean fold changes (**E**) relative to concentrations at HC fortified milk at baseline (‘0’). The x-axis shows time from baseline (‘0’) to 5 h (‘5’), for each formula (HC, WG, WC). White represents a 0 log fold change from hydrolysed cow fortified milk at baseline. Red represents a 1 log fold increase; purple represents a 2 log fold increase; green represents a 1 log decrease. There were time × treatment interactions for all amino acid groupings (TAA, BCAA, EAA, NEAA; *p* < 0.001 each, respectively; Sidak corrected post hoc tests). Φ denotes statistical significance *p* < 0.05 between WG and HC fortified milks; χ *p* < 0.05 between WC and HC fortified milks (Sidak corrected post hoc tests).

TAA, BCAA, EAA, and NEAA plasma concentrations peaked 60 min after ingestion for all fortified milks (*p* < 0.001), and subsequently declined throughout the intervention (300 min). Most apparent was the accelerated reductions in TAA, BCAA, and EAA for the HC milk, relative to WC and WG (Figure 2A–C). Individual amino acid responses are shown in Figure 2E. 

Alanine, lysine, and proline demonstrated the greatest increase relative to baseline concentrations; of these, lysine and proline postprandial concentrations differed between the HC and both the WC and WG drinks (interaction time × treatment; *p* < 0.001 each, respectively). The difference for proline reflected the lower proline content of the whey-only HC fortified milk relative to the whole fortified milks (Table 2) Yet, the AUC of lysine was still greater following WG compared to HC, despite compositional differences (*p* = 0.014). 

The greatest differences in amino acid response between milks were comparisons of HC with both WG and WC. Marked suppression in the BCAA (leucine, isoleucine and valine) from 120 min after HC ingestion was evident. Notable were the markedly lower plasma valine concentrations that are reflective of the low valine content of HC fortified milk. 

HC milk resulted in significantly lower AUC for all measured proteogenic amino acids than either WG or WC except for alanine, isoleucine, glycine, and aspartic acid (Table 4; treatment effect, *p* > 0.05 each, respectively). This was despite the HC milk’s greater lysine, leucine, and threonine content relative to whole protein fortified milks (Table 2). Furthermore, unlike the whole protein fortified milks (WG and WC), the amino acid AUCs following HC milk were negative for many amino acids: leucine, isoleucine, valine, histidine, methionine, arginine, serine, tyrosine, and glutamic acid. 

WG and WC AUC differed only for valine and tyrosine; with valine the AUC for WG was greater than both WC and HC (Table 4; *p* < 0.001). Yet, the plasma concentrations of valine were not significantly different between WG and WC following ingestion at any specific timepoint. In contrast, the higher tyrosine in WC resulted in a higher AUC than WG milk (*p* < 0.001). This higher tyrosine following WC was apparent as greater tyrosine concentrations than WG over the postprandial period (Figure 2E; *p* < 0.05 at each hour, respectively). Both leucine and isoleucine were more abundant in WC milk than WG, which coincided with greater postprandial concentrations of each (*p* < 0.01 WC vs. WG at 1 h for leucine, *p* < 0.01 WC vs. WG at 3 and 4 h for isoleucine, each, respectively). However, these differences were less pronounced than when compared with leucine and isoleucine-rich HC milk. Threonine, although more abundant in WG, was reduced more in plasma following WG than WC milk (*p* < 0.01 WG vs. WC at 3 h); yet, AUC was not different (*p* > 0.05).

### 3.3. Postprandial Glycaemic Response

**Figure 3:**
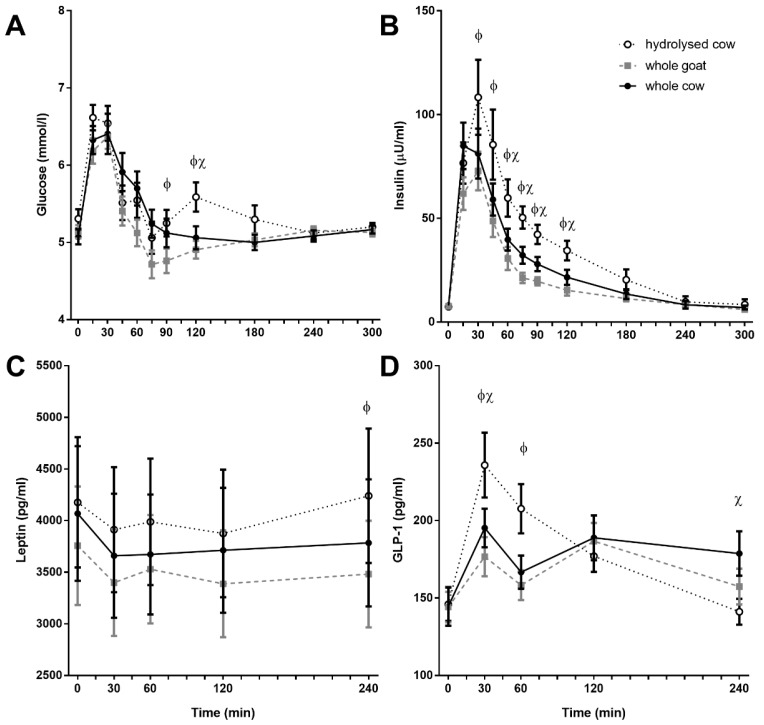
Postprandial plasma responses for glucose (**A**), insulin (**B**), leptin (**C**), and active GLP-1 (**D**). Values represent means ± SEM in mmol/L for glucose, μU/mL for insulin, pg/mL for leptin, and glucagon-like peptide-1 (GLP-1). There was a time × treatment interaction for glucose, insulin, leptin, and GLP-1 (*p* < 0.001, *p* < 0.001, *p* < 0.05, and *p* < 0.001 respectively). Φ denotes statistical significance *p* < 0.05 between whole goat (WG; ■) and hydrolysed cow (HC; ○) fortified milks; χ *p* < 0.05 between whole cow (WC; ●) and HC fortified milks (Sidak corrected post hoc tests).

All fortified milks resulted in a transient increase in blood glucose that tended to return to fasting levels by 75 min post-ingestion. HC exhibited a rebound rise in plasma glucose at 90 and 120 min (*p* < 0.05 compared to WG at both time points; *p* = 0.008 compared to WC at 120 min; Figure 3A).

### 3.4. Plasma Hormone Response

HC resulted in an increased insulin response, relative to both WC and WG, with greater insulin concentrations from 30 to 180 min post-ingestion (*p* < 0.05 between each formula, respectively; Figure 3B). Insulin AUC was greatest after HC, followed by WC and WG (8089 ± 1396, 5202 ± 788, and 3796 ± 518 µU·min/mL, respectively; interaction time × treatment; *p* < 0.001; no figure shown). Baseline plasma leptin concentrations were greater in female subjects (5472 ± 205 vs. 1965 ± 132 pg/mL in males, *p* < 0.001), although postprandial responses did not differ between sexes. Plasma hormone responses to milks tended to be similar with both WC and WG. Differences were present following ingestion of HC fortified milk, with higher leptin at 240 min (compared to WG; *p* = 0.038; Figure 3C) and higher GLP-1 at 30 and 60 min (compared to both WC and WG; *p* < 0.05 each, respectively; Figure 3D). Neither ghrelin nor CCK differed between fortified milks (data not shown).

### 3.5. Plasma Paracetamol Recovery

**Figure 4:**
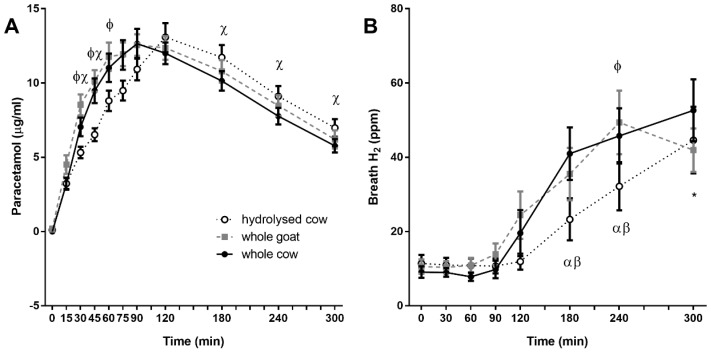
Postprandial plasma paracetamol (**A**) and breath hydrogen (**B**) responses. Values represent means ± SEM in μg/mL (**A**) and ppm (**B**). There was a time × treatment interaction for postprandial plasma paracetamol concentrations (*p* < 0.001) and breath hydrogen (*p* < 0.05). Φ denotes statistical significance *p* < 0.05 between whole goat (WG; ■) and hydrolysed cow (HC; ○) fortified milks; χ *p* < 0.05 between whole cow (WC; ●) and HC fortified milk; α *p* < 0.05 from baseline for WC fortified milk; β *p* < 0.05 from baseline for WG fortified milk; * *p* < 0.05 from baseline all fortified milks (Sidak corrected post hoc tests).

Plasma paracetamol recovery differed significantly between fortified milks (time × treatment interaction *p* < 0.001). WC and WG milks exhibited a similar response curve, whilst paracetamol recovery was delayed with HC ingestion for the first 90 min post-ingestion (Figure 4A). Total paracetamol recovery (AUC), did not differ between fortified milks (HC: 2742 ± 163, WC: 2816 ± 234, and WG: 2861 ± 187 mmol·min/mL, respectively, *p* = 0.724).

### 3.6. Breath Hydrogen

Changes in breath hydrogen concentrations differed significantly between fortified milks (time × treatment interaction *p* = 0.016, Figure 4B). WC and WG fortified milks showed a similar response curve, while increases in HC breath hydrogen were delayed (*p* = 0.046 between WG and HC at 240 min, *p* = 0.970 and *p* = 0.222 between WG and WC, WC and HC, respectively).

### 3.7. Digestive Comfort and Appetite Scores

**Figure 5:**
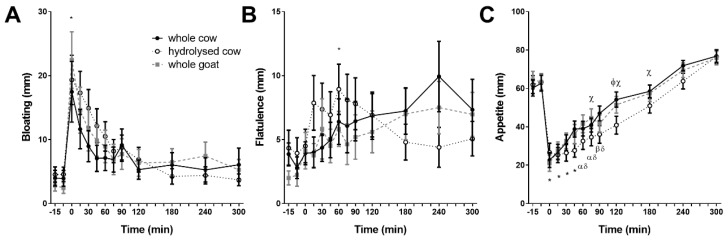
Postprandial subjective digestive symptom and appetite responses. Bloating (**A**), flatulence (**B**), and appetite (**C**). Values represent means ± SEM on a 100 mm scale. Symptom scales were anchored with 0 “no symptom” and 100 “the most severe symptom imaginable”. Appetite was anchored with 0 “nothing at all” and 100 “a lot” in response to “How much do you think you can eat?”. There was a time × treatment interaction for appetite (*p* < 0.001). Φ denotes statistical significance *p* < 0.05 between whole goat (WG; ■) and hydrolysed cow (HC; ○) fortified milks; χ *p* < 0.05 between whole cow (WC; ●) and HC fortified milks; * *p* < 0.05 from baseline for all fortified milks; α *p* < 0.05 from baseline for WC fortified milk; β *p* < 0.05 from baseline for WG fortified milk; δ *p* < 0.05 from baseline for HC fortified milk (Sidak corrected post hoc tests).

Digestive comfort scores did not differ between fortified milks. *Bloating* and *flatulence* scores increased after ingestion, regardless of milk type (time effect, *p* < 0.001 and *p* = 0.009, respectively; Figure 5A,B). No other symptom scores differed (data not shown).

Appetite scores differed between fortified milks (interaction time × treatment; *p* = 0.025; Figure 5C). The HC suppressed appetite relative to baseline continuously until 90 min (*p* < 0.05), WC continuously until 75 min (*p* < 0.05) and WG until 45 min, and again at 90 min (*p* < 0.05, each respectively). *Appetite* scores were lower after HC between 120 and 180 min (*p* = 0.024 between HC and WC at 75 min and *p* = 0.012 and *p* = 0.035 from 120 to 180 min, respectively; *p* = 0.030 between HC and WG at 120). Scores for *hunger*, *fullness*, and desire to eat *sweet*, *salty*, *savoury*, or *fatty* food were not different between fortified milks (data not shown). 

## 4. Discussion

The digestive, metabolic, and subjective analysis of digestive responses following ingestion of fortified milks, formulated with either whole milk proteins (bovine or caprine) or partially-hydrolysed bovine whey protein, were analysed in healthy young adults. In contrast to the hypothesis, it was demonstrated that irrespective of the originating *bovidae* species, whole protein fortified milks were digested and metabolised similarly. Thus, amino acid appearance and the hormonal responses influenced by protein digestion, including GLP-1, CCK, and insulin, did not differ markedly between these whole protein fortified milks. Across most measures of digestion and hormone concentrations, partially-hydrolysed bovine fortified milk led to markedly different digestive responses. The response to partially-hydrolysed bovine fortified milk included an exaggerated insulin response with markedly suppressed BCAA appearance in plasma. No difference in subjective comfort was reported between the fortified milk drinks. 

Despite the differences in amino acid composition among the WG, WC, and HC fortified milks in the current study, the most substantial difference in amino acid response was observed between the partially-hydrolysed relative to whole protein fortified milks. After 1 h, the circulating AA concentrations after HC fortified milk were suppressed below fasting levels at all further timepoints. These lower concentrations of circulating AAs are either due to a delayed rate of digestion or increased clearance from the plasma. Paracetamol appearance, a proxy measure of gastric emptying [30] and with known limitations in describing transit [27], demonstrated a possible slowed rate of gastric emptying for HC relative to WC and WG. While the drinks were matched for protein content, they were not isoenergetic. It is likely that the higher caloric density of the partially-hydrolysed drink contributed to the delay in gastric emptying [35]. Further, the structural differences of the whole proteins compared to the partially-hydrolysed proteins may have also contributed to altered gastric emptying between fortified milk drinks [23]. Yet, it would be expected that the clotting properties of casein compared to whey protein would rather delay gastric emptying [23] in the whole protein fortified milk. In addition, the structural differences of protein hydrolysation have been shown not to impact gastric emptying in isoenergetic comparisons [35]. However, this difference is unlikely to account for the substantial differences in circulating AA. Interestingly, the current findings contrast with findings of increased gastric emptying following hydrolysed formulations in infants with or without gastroesophageal reflux disorder [36], others have reported slower gastric emptying with hydrolysed formulas, and there is no clear consensus [27].

In the present study, an exaggerated incretin response (e.g., GLP-1 and insulin) was demonstrated, particularly 1 h after the HC milk ingestion, relative to the whole protein milks. Insulin is a potent regulator of amino acid transporter activity [37]; therefore, these results are suggestive of greater AA disposal with HC, relative to WC and WG. It has been previously demonstrated that gavage administration of hydrolysed whey, relative to whole whey protein, elicits greater appearance of di- and oligo-peptides, which may further account for a proportion of the measurable differences in plasma AA [38]. Whether these differences in digestion and circulating AA concentrations impact on major pathways of protein metabolism, including synthesis and oxidation, is yet to be explored.

The digestive responses of gastric emptying rates (paracetamol recovery) and gastrointestinal transit (breath hydrogen) following ingestion of the whole protein fortified milks (goat and cow) were very similar. Whole goat and cow fortified milk also elicited similar satiety scores and appetite hormone levels; this aligns with reports of similarities between goat and cow dairy by others [39]. Although the increasing breath hydrogen after whole protein formulas may be expected to correlate with increasing digestive discomfort (i.e., bloating or flatulence), this was not the case. For this study, all participants self-reported dairy tolerance, and this low level of malabsorption, originating from the lactose and/or the added inulin or inositol, may have been insufficient to evoke any symptoms [40]. These similarities in gastric transit in the whole protein fortified milks occurred despite the known differences in proteins. This includes differing casein profiles which would be expected to lead to softer gastric curd formation in the goat drink [41]. Whilst curd formation may slow gastric emptying [23], other studies have failed to demonstrate this [42,43]. 

This study was conducted in adults, and while adult and infant digestion differ regarding gastric emptying, digestive enzyme production, and gastric pH [44], aspects such as enzymatic secretions and hepatic metabolism are reported to reach a comparable adult capacity by 3 to 6 months of age [44,45]. Hence, these findings should be applicable to children above the age of 12 months, for whom fortified milks are designed. It is unknown what the possible long-term impact of such short-term metabolic differences between partially-hydrolysed and whole protein formulas may be for a growing child. Delayed gastric emptying and increased satiety from partially-hydrolysed proteins might impact on appetite cues at subsequent meals. Reduced formula consumption has previously been reported in infants fed hydrolysed whey formula [46]. Alterations in nitrogen balance [47] and higher blood urea nitrogen [48,49] have been observed in infants fed various hydrolysate formulations. In the current study, the partially-hydrolysed formula differed most with a suppressed availability of branched chain amino acids, a class of amino acids with important roles in growth [50]. This is despite the comparable BCAA in the whey-exclusive hydrolysed fortified milk. However, the impacts of these postprandial alterations in amino acid availability require further investigation.

Goat milk-based products are increasingly seen as a possible alternative to cow milk-based products. The composition of goat milk may be more similar to breastmilk for components such as amino acids [51] and nucleotides [52]. Importantly, goat milk formula performs similarly to cow milk formula, supporting infant growth and development in animal and human studies. Animal studies have shown goat milk to enhance the utilisation and retention of protein [53,54], mineral absorption and tissue storage (including calcium [55,56] and iron [56,57,58]), and intestinal fat absorption [59] as compared to cow milk. Randomised clinical trials in infants show the growth curves of children exclusively fed goat milk formula are similar to both breastmilk fed infants [60] and those fed cow milk formula [60,61,62]. This has been extended to demonstrate similarity in the composition of the gut microbiota [63]. The current study builds on recent comparisons of digestive responses to goat and cow dairy meals in humans [39], and provides the first evidence in humans of a comparable digestive and metabolic response to fortified milk drinks manufactured from whole goat and cow milk. 

The current study did not find differences in digestive comfort between milks. However, since the subjects were not known to have dairy intolerance, this may have limited the detection of digestive tolerance differences, as significant intolerance would not be expected. This study was limited by the use of adult subjects and the inconsistent formulation in terms of energy, macronutrients, and ingredients. The HC milk had a greater caloric load, known to delay gastric emptying [35], while the high BCAA content of the whey may have elevated insulin secretion [64,65] relative to WG and WC milks. Further differences in carbohydrate and fat content, and composition and micronutrient composition. may have equally impacted incretin responses and gastric emptying. These differences may have influenced the current findings, and as such, deserve consideration in the context of fortified milk formulations.

## 5. Conclusions

The current study has demonstrated that fortified goat milk, compared to fortified cow milk, is digested and metabolised similarly, despite inherent compositional differences. In contrast, partially-hydrolysed fortified milk elicited marked differences in postprandial hormonal stimulation, resulting in a suppression of amino acids in circulation for the period after the first hour of ingestion. Given the importance of circulating amino acids and incretins in the regulation of appetite [24] and anabolism [66], these differences may impact feeding behaviour or have important metabolic consequences. Given these differences in the digestive and metabolic responses to partially-hydrolysed whey fortified milk, further studies are warranted to address both the impact that this may exert on appetite and protein metabolism.

## Figures and Tables

**Table 1:** Nutritional composition per 100 mL prepared fortified milk.

Nutrient	Unit	Whole Goat	Whole Cow	Partially Hydrolysed Cow ^†^
Energy	kJ	255	230	360
Protein	g	2.2	2.2	2.2
Fat	g	3.1	2.1	3.6
Total carbohydrates	g	6.1	7.0	11.0
Lactose	g	3.6	3.2	4.4
Maltodextrin	g	1.7	2.9	6.6
Fructose	g	0.6	0.7	0.0
Fibre (inulin + oligofructose)	g	0.2	0.3	0.0
Sodium	mg	23	19	35
Vitamins				
Total vitamin A (RE)	mcg	63	63	41
Vitamin D_3_	mcg	1.0	1.0	1.0
Vitamin E (TE)	mg	1.6	1.6	1.4
Vitamin C	mg	9	9	9
Thiamine	mcg	62	69	116
Riboflavin	mcg	120	137	193
Niacin	mg	0.69	0.8	0.6
Folic acid	mcg	12	12	21
Minerals				
Calcium	mg	103	94	122
Phosphorus	mg	77	73	68
Magnesium	mg	10	6.7	32
Iron	mg	1.0	1.0	1.3
Zinc	mg	0.52	0.50	0.6
Iodine	mcg	10	9	14
Other				
Inositol	mg	7.4	8.3	
Probiotic cultures				
*Lactobacillus and Bifidobacterium*	CFU (million)	40	40	40

CFU, colony-forming units; RE, retinol equivalents; TE, tocopherol equivalents. ^†^ Formula was prepared to provide 2.2 g protein per 100 mL; more powder per 100 mL was added than prescribed by the manufacturer to match protein concentration of other formulas. Nutrient values are taken from the product tin as purchased in New Zealand.

**Table 2:** Total amino acid profile per 100 mL prepared fortified milk.

Amino Acid ^†^	Whole Goat	Whole Cow	Partially Hydrolysed Cow
	mg		
Glutamic Acid	470	510	393
Proline	235	230	120
Leucine	214	232	249
Lysine	174	186	196
Aspartic Acid	161	181	247
Valine	153	151	126
Serine	123	111	94.1
Threonine	115	102	142
Phenylalanine	109	117	77.8
lsoleucine	103	123	125
Tyrosine	77.5	106	60.1
Alanine	69.6	88.8	110
Arginine	62.3	79.6	55.8
Histidine	61.9	66.3	43.4
Methionine	52.4	63.3	47.9
Glycine	38.9	45.6	43.2
Tryptophan	32.1	34.8	47.5
Cysteine	21.2	20.2	63.7

^†^ Results for aspartic acid and glutamic acid may include contributions of asparagine and glutamine, respectively, converted during hydrolysis.

**Table 3:** Baseline subject characteristics.

Measure	Unit	Males (*n* = 15)		Females (*n* = 15)	
		Mean	SEM ^†^	Mean	SEM ^‡^
Age	years	25.3	2.8	24.8	2.2
Weight	kg	74.8	10.1	58.3	7.7 ***
Height	cm	175.0	4.0	164.0	4.4 ***
BMI	kg/m^2^	24.3	2.9	21.7	2.7 *
Glucose	mmol/L	5.4	0.6	5.0	0.4 **
Insulin	μU/mL	7.3	0.8	7.9	0.4
HOMA-IR		1.8	1.3	1.7	0.6

^†^ Values presented as means ± SEM from fasting samples collected on all three occasions; ^‡^ Main effects and interactions were analysed by two-factor repeated-measures ANOVA (treatment and sex) with Sidak corrected post hoc tests. There were no differences between group baseline values between treatment days; * *p* < 0.05, ** *p* < 0.01, *** *p* < 0.001 compared with male subjects.

**Table 4:** Amino acid AUC following whole goat, whole cow, and partially-hydrolysed cow fortified milk.

Amino Acid ^†^	Whole Goat	Whole Cow	Partially Hydrolysed Cow	*p* Value ^‡^	Post Hoc
Alanine	15392 ± 2104	19273 ± 2486	18960 ± 2475	0.261	
Lysine	8768 ± 807	7837 ± 852	5903 ± 786	0.036	*
Proline	15374 ± 848	16301 ± 1105	2613 ± 973	<0.001	***^^^
Leucine	1771 ± 565	1344 ± 791	−180 ± 547	0.03	**
Isoleucine	46 ± 300	558 ± 554	−304 ± 358	0.219	
Valine	6138 ± 725	1676 ± 905	−6833 ± 795	<0.001	***^^^###
Tyrosine	489 ± 244	2237 ± 334	−2395 ± 303	<0.001	***^^^###
Methionine	51 ± 115	255 ± 153	−914 ± 126	<0.001	***^^^
Phenylalanine	−208 ± 198	−77 ± 189	−2388 ± 226	<0.001	***^^^
Asparagine	1095 ± 197	1300 ± 207	384 ± 242	0.003	*^
Citrulline	3204 ± 471	1377 ± 443	2609 ± 582	0.019	##
Glutamine	9639 ± 1514	5988 ± 1758	1146 ± 1674	<0.001	**
Ornithine	1108 ± 332	791 ± 410	−636 ± 305	<0.001	***^
Histidine	1579 ± 398	1616 ± 453	−1162 ± 322	<0.001	***^^^
Serine	326 ± 371	−38 ± 435	−1646 ± 385	0.003	**
Taurine	−3644 ± 2665	−1634 ± 1671	−1025 ± 692	0.566	
Aspartic Acid	−30 ± 92	−59 ± 106	−172 ± 38	0.211	
Glutamic Acid	518 ± 517	652 ± 922	−1347 ± 617	0.027	*
Glycine	−2423 ± 731	−2665 ± 666	−3877 ± 732	0.26	
Hydroxy-proline	−208 ± 53	−330 ± 85	−292 ± 69	0.359	
Arginine	1250 ± 725	1167 ± 706	−2431 ± 604	<0.001	**^^
Threonine	−1108 ± 172	−1299 ± 173	−2160 ± 149	<0.001	***^^^

^†^ Values presented as means ± SEM in μmol·min/mL; ^‡^ Main effects and interactions were analysed by one-factor repeated-measures ANOVA (treatment) with Sidak corrected post hoc tests. * *p* < 0.05, ** *p* < 0.01, *** *p* < 0.001 between whole goat and partially-hydrolysed cow fortified milk; ^ *p* < 0.05, ^^ *p* < 0.01, ^^^ *p* < 0.001 between whole cow and partially-hydrolysed cow fortified milk; ## *p* < 0.01, ### *p* < 0.001 between whole goat and whole cow fortified milk.
